# Uptake of the antisecretory factor peptide AF-16 in rat blood and cerebrospinal fluid and effects on elevated intracranial pressure

**DOI:** 10.1007/s00701-014-2221-7

**Published:** 2014-09-24

**Authors:** Mohamed Al-Olama, Stefan Lange, Ivar Lönnroth, Kliment Gatzinsky, Eva Jennische

**Affiliations:** 1 Institute of Biomedicine, Sahlgrenska Academy, University of Gothenburg, P.O.B. 440, 405 30 Göteborg, Sweden; 2 Department of Neurosurgery, Sahlgrenska University Hospital, 413 45 Göteborg, Sweden

**Keywords:** AF-16 uptake, Intranasal administration, Brain oedema, Intracranial pressure, Telemetry, Continuous recording, Rat

## Abstract

**Background:**

AF-16 is a 16-amino-acid-long peptide derived from the amino-terminal part of the endogenous protein, antisecretory factor (AF). AF-16 in vivo has been shown to regulate dysfunctions in the water and ion transport system under various pathological conditions and also to counteract experimentally increased tissue pressure.

**Methods:**

Rats were subjected to a cryogenic brain injury in order to increase the intracranial pressure (ICP). The distribution of AF-16 in blood and CSF after intravenous or intranasal administration was determined in injured and control rats. ICP was monitored in freely moving, awake rats, by means of an epidural pressure transducer catheter connected to a wireless device placed subcutaneously on the skull. The continuous ICP registrations were achieved by means of telemetry.

**Results:**

Intranasal administration of AF-16 resulted in a significantly higher CSF concentrations of AF-16 in injured than in control rats, 1.3 versus 0.6 ng/ml, whereas no difference between injured and control rats was seen when AF-16 was given intravenously. Rats subjected to cryogenic brain injury developed gradually increasing ICP levels. Intranasal administration of AF-16 suppressed the increased ICP to normal values within 30 min.

**Conclusion:**

Optimal AF-16 concentrations in CSF are achieved after intranasal administration in rats subjected to a cryogenic brain injury. The ability of AF-16 to suppress an increased ICP was manifested.

## Introduction

A pathological rise in intracranial pressure (ICP) may occur as a response to major stroke and trauma. Patients affected by these conditions are most often subjected to an increased acute mortality and long-term morbidity with significant neurological deficits [1–3]. AF-16, a 16-amino-acid-long peptide derived from the amino-terminal part of the endogenous protein, antisecretory factor (AF), [4, 5] reduces high ICP in rats with experimentally induced herpes type 1 virus encephalitis [6]. Preliminary observations indicate that AF-16 also suppresses high ICP resulting from focal cryogenic brain injury [7]. Further investigation of AF-16 as a putative regulator of ICP is therefore merited.

Previous studies have demonstrated suppressive effects of AF-16 on increased tissue pressure in tumours [8]. The modulating influence of AF-16 under these conditions is achieved after intravenous (i.v.) or intranasal administration. Measurements of the therapeutic concentrations of AF-16 have, however, not been made. In the present study the AF-16 concentrations in plasma and cerebrospinal fluid (CSF) were determined after intranasal and after i.v. administration of the peptide in control rats and in rats subjected to a focal cryogenic brain injury.

In a separate set of experiments, the effects of AF-16 on ICP were recorded. A number of methods for measuring ICP in anaesthetised rats have been described, including epidural monitors, subdural, ventricular and cisterna magna catheters, intra-parenchymal transducers and, recently, lumbar cannulation (for a comprehensive overview see Uldall et al. [9]). These techniques provide relevant data, but during the registrations the central nervous system (CNS) tissue is exposed to anaesthetic drugs which might affect ICP recordings. In addition, registration of ICP mainly with intra-parenchymal and intra-ventricular probes usually gives higher and more fluctuating values than probes placed epidurally, and leads to an increased risk of intra-parenchymal haematoma and hydrocephalus [9].

Silasi et al. [10] were the first to use telemetry blood pressure transmitters to monitor the ICP in awake rats. The technique they used, based on a method developed by Data Science International (DSI), was complicated. In the present study, we present a modified method for continuous, long-term measurement of ICP by the DSI technique in order to allow recording of AF-16 effects in un-anaesthetised and freely moving rats. Recordings were made through a pressure-sensitive transducer catheter inserted epidurally and connected to a small device which is radiometrically connected to a computer system.

In summary, the results show that the concentration of AF-16 in plasma is about ten-times higher than in CSF after both i.v. and intranasal administration of the peptide. Intranasal administration gave a significantly higher CSF concentration of AF-16 in animals subjected to a cryogenic brain injury compared with uninjured controls. Furthermore, AF-16 administered intranasally suppressed increased ICP in the injured animals in a significant manner.

## Materials and methods

### Animals

The test protocol was approved by the Regional Animal Experiments Ethical Committee and performed in accordance with guidelines for animal experiments (EC Directive 86/609/EEC). Male Sprague–Dawley rats, 180 ± 20 g body weight (Nova-SCB, Sollentuna, Sweden) were used. The rats were allowed a week for general adaptation in their cages before any form of experimental procedure was performed. All rats had free access to water and pelleted food during the whole experimental period. The temperature and air ventilation in the animal quarters were monitored according to standard procedures.

### Chemicals

AF-16, produced by organic solid phase synthesis, was purchased from Ross-Pedersen (K.J. Ross-Pedersen ApS, Klampenborg, Denmark). The peptide was purified by reversed-phase chromatography and analysed by amino acid analysis and mass spectrometry. The purity of AF-16 was about 97 %. The AF-16 peptide, VCHSKTRSNPENNVGL, was labelled with ^14^C in the N-terminal valine, specific activity 50 mC/mmol, 2 μM/ml (American Radiolabeled Chemicals, Saint Louis, Mo, USA). Marcaine® was purchased from AstraZeneca (Södertälje, Sweden), Isoflurane® from Baxter Medical (Kista, Sweden).

### Surgical procedure for cryogenic brain injury

The operation procedure started with general anaesthesia via inhalation of Isoflurane (4 % induction, 1.5–2 % maintenance in air), after which the anaesthetised rat was positioned on the abdomen on a heating pad. The body temperature was monitored via a rectal thermo-coupled probe, and a heated blanket was placed underneath the rat in order to keep the temperature at 37 °C. The head was shaved and the skin washed with 70 % alcohol, followed by a subcutaneous injection of Marcaine. A 3– to 4-cm-long midline incision was made through the skin and fascia on the skull vault. The skull bone was freed from adherent connective tissue and a copper rod (144 g) with a tip diameter of 4 mm was immersed in liquid nitrogen and then applied to the right parietal bone for 90 s. This freezing procedure penetrated the bone and induced a defined morphological injury in the underlying brain tissue The midline of the cranium was avoided in order not to induce any damage to the superior sagittal sinus. The resulting lesion volume was calculated after termination of the experiments (see below). Uninjured rats were used as controls.

### Preparation for continuous ICP recordings

**Fig. 1:**
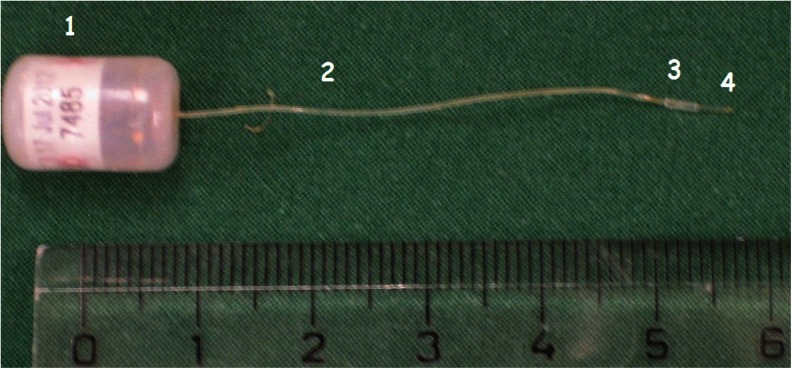
The various parts of the device for ICP recording: pressure transducer (*1*), transducer catheter (*2*), plastic cannula (*3*) and tip of the sensor probe (*4*)

The pressure transducer catheter (PA-C10, Data Sciences International, St. Paul, MN, USA) was inserted into a plastic cannula. The plastic cannula was 3 mm long with an inner diameter of 0.40 mm and an outer diameter of 0.80 mm (Intramedic Polyethylene Tubing, BD, Franklin Lake, NJ, USA). The pressure devices were calibrated before shipping. Before implantation the calibration of each probe was verified according to the instructions given by the DSI manual. The individual components of the system for ICP recording are shown in Fig. 1.

### Implantation of ICP recording probe

**Fig. 2:**
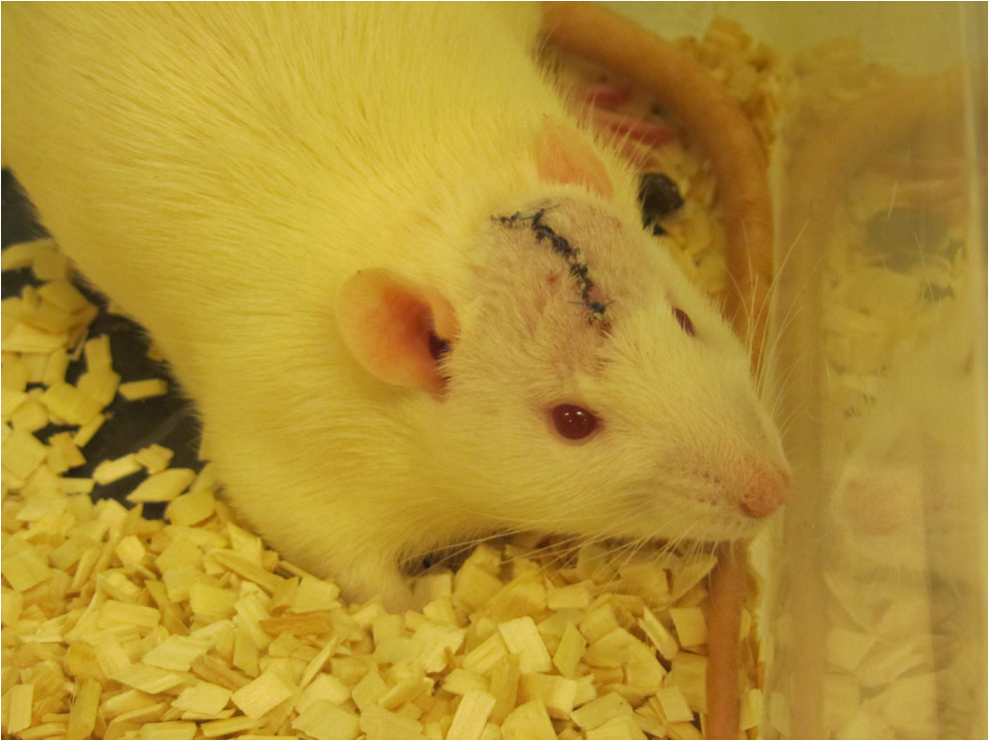
Freely moving rat with the measuring device on the skull

After imposing the cryogenic brain injury on the right hemisphere, a hole (diameter 1 mm) was drilled on the right side of the parietal bone 3–4 mm lateral to the midline and 2 mm posterior from the skull bregma mark, followed by free-dissecting the dura from the bone 3–5 mm anteriorly to the hole. The tip of the sensor probe, which was connected directly to the wireless transducer, was inserted anteriorly into the epidural space with the cannula covering the burr hole. Prior to implantation, the offset reading of each probe was recorded, and this value was subtracted from the ICP recordings throughout the experiment. Dental cement was applied around the cannula in the burr hole in order to fixate the probe to the bone and avoid air leakage into the epidural space. The transducer device was placed under the head skin, which was then closed by ethilone sutures. After completion of surgery, the rat recovered in its cage, which was placed on top of a receiver (Model RPC-1; Data Sciences International, New Brighton, UK) connected to a computer equipped with dataquest A.R.T. 2.2 software (Data Sciences International, New Brighton, UK). ICP could then be continuously monitored in the awake and drug-free rat for the rest of the experimental period. A freely moving rat with an implanted device is shown in Fig. 2.

### Intranasal administration of AF-16

Rats were anaesthetised with Isoflurane 24 h after the cryogenic brain injury. The rats were given a single dose of 25 μl of AF-16 (2,000 μg/ml in phosphate buffered saline, PBS) or ^14^C-AF-16 in each nostril and were then placed on their backs to make sure the AF-16 solution was absorbed appropriately. After 5–8 min they had recovered from anaesthesia, were fully awake and moved freely in the cage. Control, injured rats received the same volume of vehicle (PBS) only. ICP recordings for statistical calculations were collected for a 20-min-long period, starting after the animals were fully awake and the ICP values were stable and back to pre-anaesthesia levels.

### Calculation of lesion volume and histology

At the end of the experiments the rats were again anaesthetised, the thorax opened, vena cava inferior cut open and the rats were terminated by bleeding. The skull was opened, and the brain carefully dissected out and placed into 4 % buffered formaldehyde for about 1 week. The lesion volume resulting from the cryogenic injury was estimated by measuring the radius of the damaged area on the surface of the fixated brain with the aid of a calliper and using the formula: 2 V = 4/3 × π × *r* ^*3*^. We assumed that the injured area represented half a sphere. In order to visualise the histology of the lesion, five brains were embedded in paraffin, sectioned and stained with cresyl violet, using standard procedures.

### Blood and CSF sampling

The collection of blood and CSF was done in a separate set of experiments. ^14^C-Labelled or unlabelled AF-16 was given by intranasal (see above) or i.v. administration via the penis vein (25 μl in a volume of 0.5 ml). For measurement of blood concentration of AF-16 the heart was punctured from the right thoracic cavity with an O.D. 1.22/60 mm injection needle. When 10–12 ml of blood had been collected, the thorax was opened and the rats were terminated by removing the heart. Immediately after termination, the rats were placed on the abdomen. For measurement of CSF levels of AF-16 the dura over cisterna magna was micro-surgically exposed by dissecting the neck muscles laterally, a procedure which uncovers the midline. The dura was subsequently punctured by a Neoflon i.v. cannula, outer diameter 0.6 mm, length 19 mm. After removing the needle from the cannula, 50–100 μl of CSF could be aspired without being contaminated with blood.

### Analyses of AF-16 in plasma and CSF

A Bachem AF-16 Enzyme Immunoassay kit (item S-1403) was used. Heparin-treated blood samples were mixed with an equal volume of Alsevers citrate solution (glucose, 0.1 M, sodium citrate 2-hydrate, 0.03 M, sodium chloride, 0.07 M). The blood samples were stored frozen for up to 1 month at −20 °C. The laboratory procedure followed Protocol III in the Bachem kit manual, but some critical points should be emphasised. The standard AF-16 was dissolved and diluted in normal blood plasma; the standards S1-S6 (0.2–200 ng/ml, corresponding to 0.114–114 nM) and normal plasma S0 were distributed into smaller aliquots (e.g. 200 μl) and frozen at −20 °C until use. The standard is not stable in buffered saline at these concentrations. After dissolving the biotinylated tracer in enzyme immunological assay buffer, the solution was used within a week or was alternatively frozen in smaller aliquots until use. After adding samples and antiserum to the wells, mixing was achieved with a pipette. After addition of 2 M HCl, mixing was avoided and the absorbance was read at 450 nm. The AF-16 levels in CSF were under the detection level of the AF-16 Enzyme Immunoassay. It was therefore estimated from the radioactivity achieved after injection of ^14^C-AF-16. The immunochemical and the radioactive method gave similar values in blood and in CSF

### Autoradiography

The distribution of ^14^C-AF-16 in the brain after intranasal administration was investigated in a separate set of experiments. Control, uninjured rats (*n* = 10) received one intranasal dose of ^14^C-AF-16. Thirty minutes later the rats were terminated and the brains were dissected out as described above. Frontal slices were cut, about 3 mm thick, immersed in 10 % sucrose in PBS overnight and then frozen in liquid nitrogen. Cryosections, cut at 10 μm, were prepared, air dried and exposed to Biomax AR autoradiography film (Kodak).

### Statistics

Statistical analyses were performed using one-way Student’s *t*-test, and a value of *p* < 0.05 was regarded as statistically significant.

## Results

### AF-16 in blood and in CSF

**Fig. 3:**
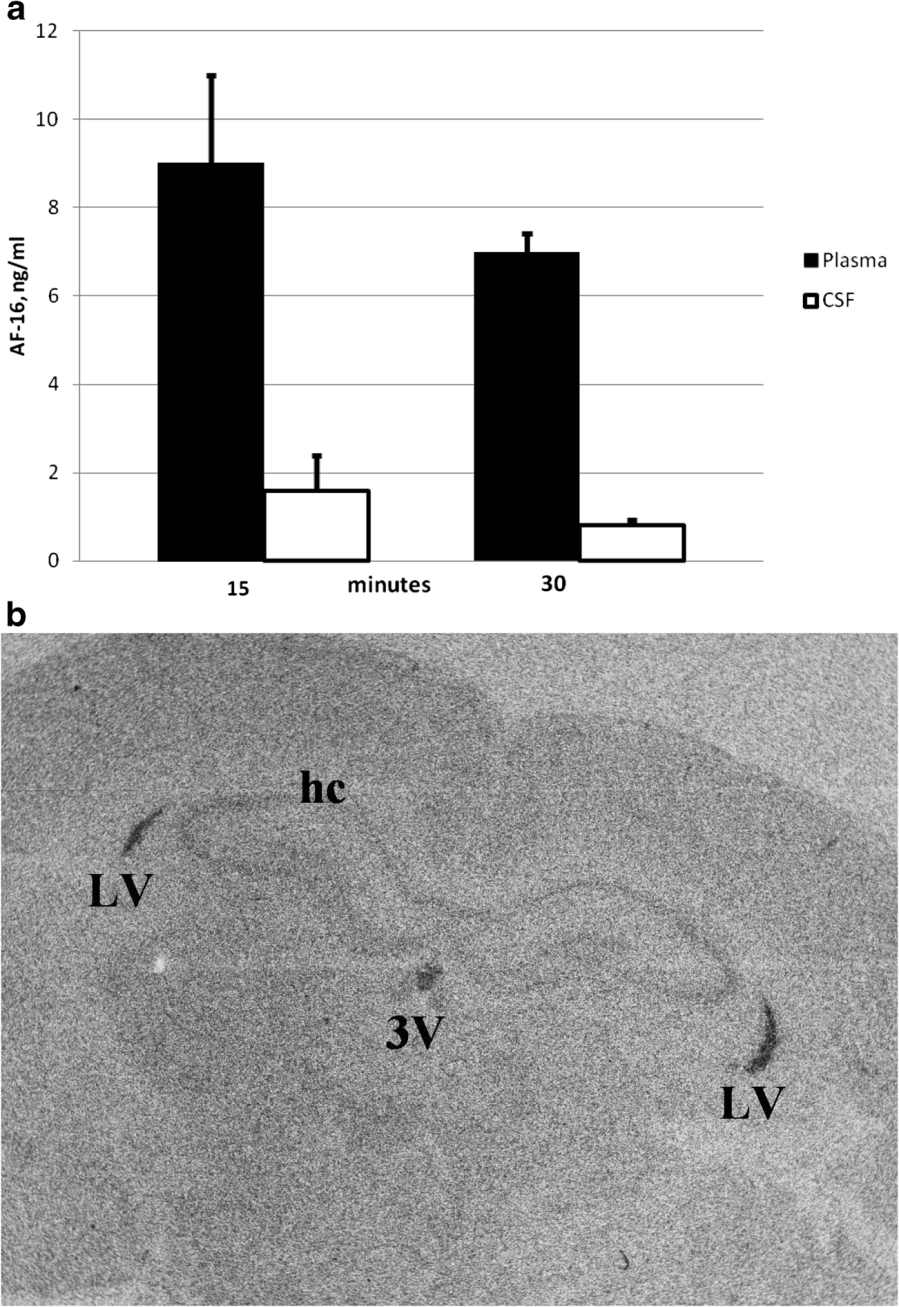
**a** AF-16 was deposited intranasally (0.3 μmol/kg in 25 μl) under Isoflurane anaesthesia, after which the concentration (ng/ml) of the peptide was determined in plasma (*filled bars*) and in CSF (*unfilled bars*) after a 15-min- or a 30-min-long period. Higher levels of AF-16 are found in plasma than in CSF at both times. Mean ± SEM. **b** Autoradiography showing distribution of ^14^C-AF-16 in the rat brain 30 min after intranasal administration of the peptide. X-ray film showing signal in the lateral (*LV*) and third ventricles (*3V*); *hc* hippocampus

Blood and CSF samples were collected 15 and 30 min after the intranasal deposition of AF-16 in normal animals and peptide concentrations were determined by enzyme-linked immunosorbent assay (ELISA) (Fig. 3a). The concentration of AF-16 was about ten-times higher in plasma than in CSF at both sampling points. The plasma levels decreased significantly between 15 and 30 min (*p* < 0.03), which indicates a rapid turnover rate of plasma AF-16. The decrease of AF-16 in CSF was not statistically significant. Autoradiography showed that ^14^C-AF-16 could be detected in the brain ventricles/choroid plexus 30 min after intranasal administration (Fig. 3b).

To compare the two different administration routes, blood and CSF samples were collected 30 min after an intranasal or an i.v. deposition of AF-16. The samples were collected both from uninjured control animals and from experimental animals subjected to cryogenic injury. In all animals the concentration of AF-16 was about ten-times higher in plasma than in CSF (Table 1). The AF-16 concentration in CSF was similar in control rats and in experimental rats after i.v. administration. Using the intranasal route, however, AF-16 administration resulted in a significantly higher CSF concentration in experimental than in control rats (*p* = 0.01; Table 1). Thus, compared with the i.v. route, the intranasal route gave a similar or higher concentration of AF-16 in the CSF. The intranasal route was therefore chosen for the following ICP experiments. This route is also a less invasive way of giving the peptide to the rats.

**Table 1:** Concentrations of AF-16 in plasma and in CSF after intravenous and intranasal administration in control and experimental rats

Group	Administration route	Fluid	*n*	Mean ± SEM (ng/ml)	*p*
Control	i.v.	Plasma	7	14.7 ± 0.6	NS
Experimental	i.v.	Plasma	7	14.5 ± 0.9	
Control	i.v.	CSF	6	1.1 ± 0	NS
Experimental	i.v.	CSF	7	1.1 ± 0.1	
Control	i.n.	Plasma	9	7.8 ± 0.7	NS
Experimental	i.n.	Plasma	9	8.7 ± 0.5	
Control	i.n.	CSF	9	0.6 ± 0.1	0.01
Experimental	i.n.	CSF	9	1.3 ± 0.2	

*Control* rats subjected to no form of treatment, *Experimental* rats subjected to acryogenic brain injury; *i.v.* intravenous administration, *i.n.* intranasal administration; *NS* not significant

### ICP measurements

**Fig. 4:**
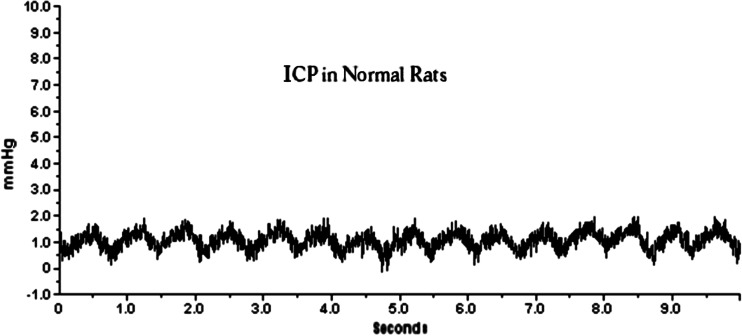
ICP recording from a control rat showing pulsations due to the heart beats

The ICP measured epidurally in normal, uninjured rats was 3.15 ± 1.4 mmHg (*n* = 10). The ICP varied with heart beat and respiration (Fig. 4). The cryogenic injury did not induce any form of neurological deficits or abnormal movement patterns in the rats. The animals were moving and feeding normally. ICP measurements collected after the cryogenic injury showed that the ICP started to increase 2–3 h after the injury, peaking between 12 and 24 h.

### Effect of treatment with AF-16 or PBS vehicle

**Fig. 5:**
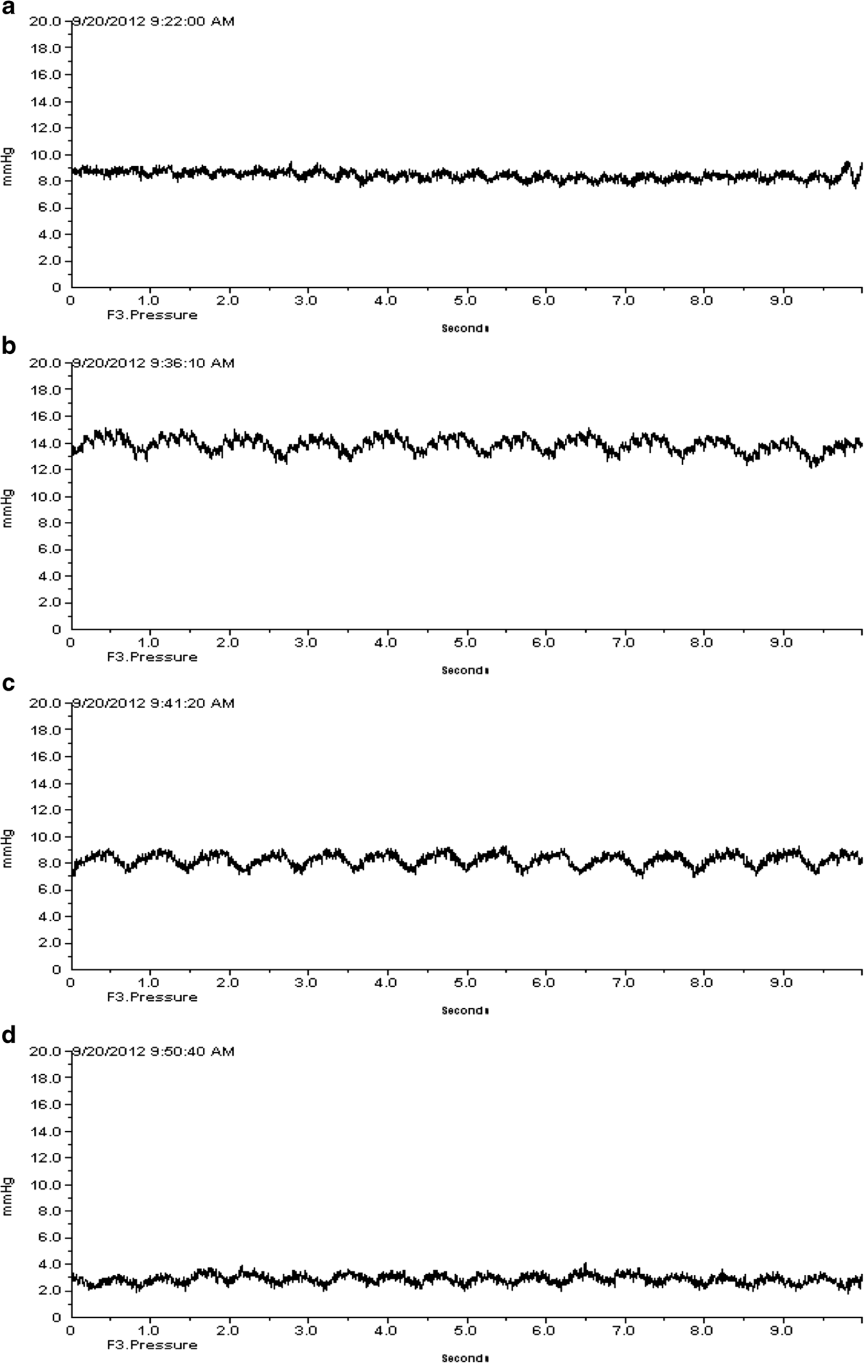
Recordings of ICP 24 h after a cryogenic injury. **a** Before anaesthesia. **b** Transient increase in ICP during induction of anaesthesia. **c** Return to the pre-anaesthetic level after a couple of minutes. **d** Reduced ICP 30 min after AF-16 administration

The induction of anaesthesia caused a transient increase in ICP (Fig. 5), but after 3–5 min ICP returned to pre-treatment levels, after which AF-16 or the vehicle (PBS) was administered intranasally. Table 2 shows the mean decrease in ICP in percent after intranasal administration of AF-16 or PBS, respectively. The decrease in ICP is calculated as the difference between the initial, pre-treatment levels and the mean of ICP recordings during the first 20-min period after the animals had fully recovered from anaesthesia. ICP decreased from initial, pre-treatment level of 10.01 ± 1.99 mmHg to 7.93 ± 1.31 mmHg in rats (*n* = 10) treated intranasally with PBS. In rats (*n* = 10) given AF-16 intranasally the ICP decreased from 10.06 ± 1.84 mmHg to 6.09 ± 1.03 mmHg. The ICP reduction was significantly larger in the AF-16-treated animals than in the PBS-treated animals (*p* < 0.002). A typical course with reduction in ICP after intranasal administration of AF-16 in an animal subjected to a cryogenic brain injury is shown in Fig. 5.

**Table 2:** Influence of intranasal administration of AF-16 or PBS on the increased ICP

Category	*n*	Mean ± SEM	Significance^a^
PBS	10	15.9 ± 4.9	*p* < 0.002
AF-16	10	38.1 ± 3.8	

Recordings were collected during a 20-min-long period in the awake animals. The means were used for statistical calculations. The figures represent ICP decrease in percent (mean ± SEM) in relation to the initial raised ICP due to a cryogenic brain injury

^a^ Student’s *t*-test, PBS versus AF-16, two-tailed

### Lesion volume and histology

**Fig. 6:**
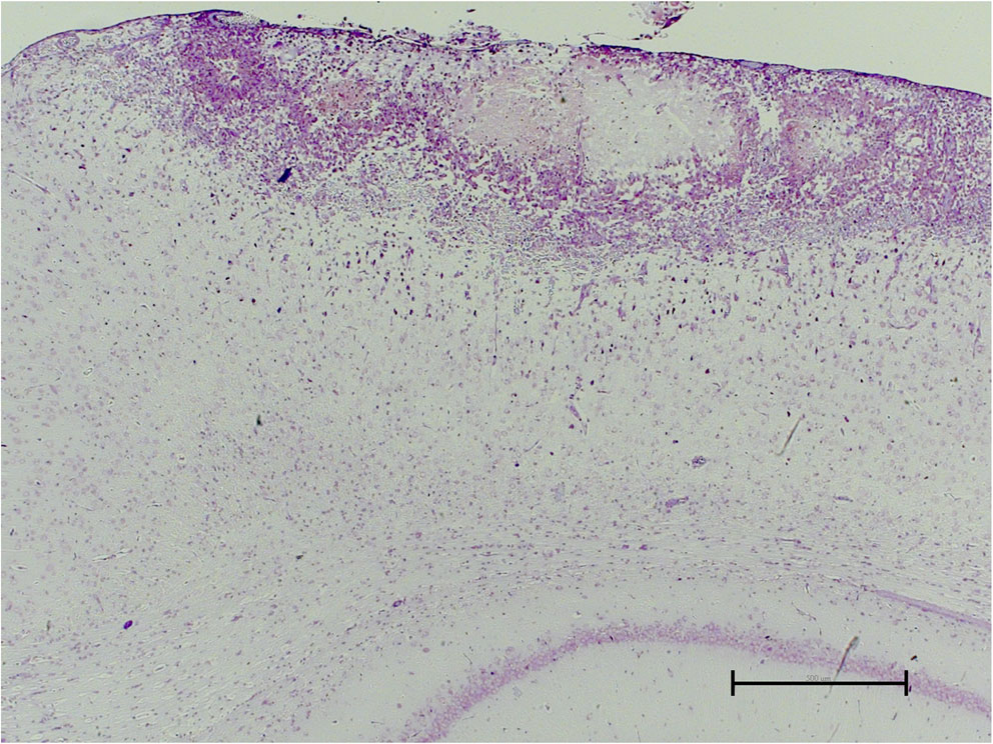
Paraffin section from the brain of an injured rat showing a typical superficial, rounded lesion. Cresyl violet. *Scale bar* 500 μm

The mean lesion volume, calculated on the assumption that the lesion was shaped as a half sphere was 12.2 ± 1.8 mm^3^ (*n* = 20). Morphological examination of the cryogenic damage to the brain showed that the injury appeared as a superficial, rounded necrosis in the parietal cortex. The histology of a typical lesion is shown in Fig. 6.

## Discussion

In the present study, we further explore the ability of AF-16 to suppress an elevated ICP utilising an experimental model of focal brain injury in rats. A significant suppressing effect on the increased ICP was seen after intranasal administration of AF-16, which reached concentration levels of about 10 ng/ml in plasma and 1 ng/ml in CSF. This effect was demonstrated using a modified method for ICP monitoring where the measuring probe was implanted epidurally and connected to a wireless device placed subcutaneously on the skull for telemetric recording. This method allows a continuous registration of ICP in the freely moving animal not subjected to influences by anaesthetic drugs. In our previous studies, ICP was measured intermittently in anaesthetised animals via a probe inserted directly into the brain tissue [6, 11, 12,] which in general gives higher resting ICP values and increases the risk of mechanical damage and infectious contamination of the brain tissue [9]. These risks are reduced considerably by the method for epidural registration used in the present study. The epidural ICP values in uninjured controls were in accordance with those reported by others [10] and, based on these data, we considered values above 6 mmHg as pathologically raised.

During induction of Isoflurane anaesthesia, a temporary increase of ICP was registered in controls as well as in experimental rats. This transient effect on ICP may be attributed to the prominent relaxation effect of Isoflurane on the musculature of blood vessels. Thus, this reaction leads to a vasodilatation with increased blood flow and cerebral perfusion [13, 14]. After autoregulatory compensating mechanisms become activated due to the increased cerebral perfusion, blood flow most likely returns to pre-anaesthetic levels, leading to a decrease in ICP.

In rats given the peptide by the intranasal route, a higher CSF concentration was obtained in animals subjected to a focal cryogenic brain injury than in control rats. This was not seen after i.v. administration. Thus, in injured rats the intranasal mode of administration appears to be more efficient than i.v. administration in order to achieve AF-16 penetration into the CNS. The major part of AF-16 recovered in blood and in CSF after intranasal administration is probably absorbed through capillaries and lymphatics in the nasal mucosa. An additional transport route into CSF after intranasal administration is through fluid-filled perineurial channels created by the olfactory ensheathing cells in the olfactory mucosa [15]. Nasal absorption via these cells has been shown to take from a few minutes up to 30 min [16, 17], a time span which is compatible with the results obtained in the present study. In addition, it is also possible that a head injury affects this route by making the epithelium more “leaky”, which might stimulate the uptake in CSF. An increased CNS uptake mediated by an enhanced axoplasmic retrograde transport is less likely, since it is a slower process which needs hours to days to make a drug reach the CNS [18].

Several studies have shown differences in uptake and effects of various drugs depending on administration route [19]. Our previous results demonstrate that i.v. administration of AF-16 was less effective than intranasal administration in reducing interstitial fluid pressure in experimentally induced mammary tumours [8]. This difference might reflect that AF-16 is more effectively bound to plasma proteins after i.v. than after intranasal deposition, and that this protein binding inhibits the subsequent clinical effects.

AF was originally identified as a potent inhibitor of pathological, enterotoxin-induced intestinal fluid secretion [5, 20]. In addition to electrolyte and water transport regulation, AF acts by counteracting various forms of inflammatory reactions [21–23]. AF has also been demonstrated to modulate the proliferation of memory/effector T cells, thereby inhibiting the severity of experimental autoimmune encephalitis [24, 25], and to inhibit the extent of inflammation in the late phase of a mouse colitis model [26]. Furthermore, we have shown that intranasal application of AF-16 suppressed an elevated ICP and completely abolished the mortality in rats with encephalitis experimentally induced by herpes simplex virus (HSV-1) [6]. The mechanisms behind the effects of AF-16 on brain tissue are, however, unclear. They may in part be ascribed to interactions with neuronal cells, since various AF peptides inhibit GABA and chloride permeation across Deiters’ neuronal cell membranes in vitro [27]. Bicuculline abrogates this inhibition, suggesting that AF interacts via the GABA_A_ receptors [27]. In addition, AF-16 also affects neuronal signalling in the brain. Thus, AF-16 incubation of acute rat hippocampal slices was followed by a 40 % reduction of GABA_A_–mediated synaptic transmission into CA1 pyramidal cells without affecting glutamatergic transmission [28]. This inhibitory effect was mimicked by feeding the rats with a diet containing specially processed cereals or after repeated cholera toxin per oral immunisations. Both of these treatments induce endogenous AF synthesis [28]. No side effects of AF-16 treatment have been observed during experimental conditions in animals.

In summary, the present study manifests the ability of AF-16 to counteract increased tissue pressure, such as seen under conditions with elevated ICP. New data are supplied on the uptake of the peptide, showing that intranasal administration is the most efficient way to achieve a fast delivery of AF-16 to the CNS. The data also indicate that the turn-over rate of AF-16 is comparatively short, suggesting that a continuous administration of the peptide would probably be optimal for keeping a raised ICP at normal levels y. The results may serve as a basis for designing studies in which the modulating ability of AF on ICP can be further exploited under clinical conditions. In future studies it will also be of advantage to use micro-dialysis to analyse the extracellular concentration of AF-16.
